# Evaluation of the House Fly *Musca domestica* as a Mechanical Vector for an Anthrax

**DOI:** 10.1371/journal.pone.0012219

**Published:** 2010-08-17

**Authors:** Antonio Fasanella, Silvia Scasciamacchia, Giuliano Garofolo, Annunziata Giangaspero, Elvira Tarsitano, Rosanna Adone

**Affiliations:** 1 Istituto Zooprofilattico Sperimentale of Puglia and Basilicata, Anthrax Reference Institute, Foggia, Italy; 2 Faculty of Agriculture, University of Foggia, Foggia, Italy; 3 Department of Public Health and Animal Sciences, Faculty of Veterinary Medicine, University of Bari, Valenzano (Bari), Italy; 4 Istituto Superiore di Sanità, Roma, Italy; National Institute of Allergy and Infectious Diseases, National Institutes of Health, United States of America

## Abstract

Anthrax is a disease of human beings and animals caused by the encapsulated, spore-forming, *Bacillus anthracis*. The potential role of insects in the spread of B. anthracis to humans and domestic animals during an anthrax outbreak has been confirmed by many studies. Among insect vectors, the house fly *Musca domestica* is considered a potential agent for disease transmission. In this study, laboratory-bred specimens of *Musca domestica* were infected by feeding on anthrax-infected rabbit carcass or anthrax contaminated blood, and the presence of anthrax spores in their spots (faeces and vomitus) was microbiologically monitored. It was also evaluated if the anthrax spores were able to germinate and replicate in the gut content of insects. These results confirmed the role of insects in spreading anthrax infection. This role, although not major, given the huge size of fly populations often associated with anthrax epidemics in domestic animals, cannot be neglected from an epidemiological point of view and suggest that fly control should be considered as part of anthrax control programs.

## Introduction

Anthrax is a human and animal disease caused by the encapsulated, large rod, and spore-forming *Bacillus anthracis*. The bacteria grow vegetatively within body tissues of the host, with sporulation occurring when vegetative organisms are exposed to the atmosphere. Animal anthrax primarily affects herbivore ruminants, such as cattle, sheep, and goats, which are the most susceptible animal hosts. The disease occurs following ingestion of soil-borne anthrax spores. Human anthrax usually results from a cutaneous infection caused from the handling of infected animal products or, in rare cases, by ingesting or inhaling spores from contaminated animal products [1]. Anthrax spores may spread within a geographic region through water, insects, wild animals, birds, and contamination from body fluids of infected animals [2], [3].

The potential role of insects in the spread of *B. anthracis* to humans and domestic animals during an anthrax outbreak has been confirmed in many studies [4]. Tabanids as well as *Stomoxys calcitrans*, *Musca domestica,* and *Calliphora erythrocephala* have been evaluated as transmitting agents [5], [6]. The house fly, *Musca domestica,* has long been considered as a potential agent for disease transmission, and bacteria have been isolated from feces, vomitus, external surfaces, and internal organs of this species [5], [7]. Structurally, the fly is well adapted for collecting pathogens. Its proboscis has a profusion of fine hairs that readily collect environmental detritus. The ability of the fly to carry anthrax bacilli from anthrax-infected flesh to wounds of healthy guinea pigs has also been demonstrated [8]. However, little quantitative information regarding the spread of anthrax cells by flies exists.

In Italy, anthrax is normally a sporadic disease [9], [10]. In 2004, there was an anthrax outbreak in the Basilicata region in southern Italy, which previously had a low prevalence of anthrax. The outbreak involved a large number of animals within a large geographic area and occurred with the span of a few weeks. In total, the epidemic killed 124 animals of different species [11]. Owing to the unusual characteristics of this epidemic, some risk factors were evaluated such as the possible transmission by insect vectors [12]. The infection evolved in different phases, and flies, whether necrophilic or hematophagic, were believed to have had a role in spreading the infection. During the first phase of this epidemic, the anthrax-infected carcasses of cattle were exposed to wild carnivores (wild boars) and insects for several days. Insects had prolonged access to the infected carcasses, and in particular, to blood effusions. Ultimately, veterinary services and the farmers removed the infected carcasses, thus reducing the level of insect contamination, and consequently, the number of animal deaths decreased [12].

In this study, *Musca domestica* was evaluated as a potential mechanical vector of anthrax to verify the possible correlation between the fly populations and anthrax epidemics, such as that of Basilicata. For this purpose, the feces and vomitus (spots) of flies previously fed on an anthrax-infected rabbit carcass or contaminated rabbit blood were examined for the presence of *B. anthracis* at different intervals from the infective feed. To our knowledge, this is the first study to evaluate the ability of spores to replicate in the gut content of insect vectors and the influence of different *ingesta* on gut replication rate.

## Materials and Methods

### Maintenance of flies

Laboratory-bred specimens of *Musca domestica* were used in this study. The flies were grown in a wooden framed cage, measuring 95×46×46 cm, covered on all sides with mosquito netting, a metal bottom, and a hole and cotton sleeves on one side for hand insertion (INFIS 2000, Italy). The flies were kept at 25°C, 65% relative humidity, and constant photoperiod (12∶12 h light:dark), and were fed *ad libitum* using thick cotton-wool pads saturated with water containing a mixture of 46% powdered milk, 46% sugar, and 8% powdered eggs.

### Ethics statement

This research was performed in accordance with the *Decreto Legislativo* No. 116/92 on animal welfare and approved by the Italian Ministry of Health.

### Fly contamination by feeding on anthrax-infected rabbit

A group of about 3000 adult flies of third generation was transferred into a cage in a Biosafety Level 3 (BL3) laboratory. The same type of cage was used as described above, except the sleeved hole for hand was replaced by a sliding metal door for the insertion of a carcass into the cage. Flies that had not eaten for 24 h were allowed to come into contact with a rabbit carcass placed into the cage. The rabbit, a New Zealand White specific pathogen-free (Harlan, Italy), was infected subcutaneously with about 40000 spores of the full virulent *B. anthracis* strain A0843, cluster A.1.a, genotype 3 [9], [13]. The rabbit was necropsied after death (48–72 h after infection) and was tested by a bacterial culture method to confirm anthrax according to the procedure of Turnbull [14].

The carcass was opened, and the gut and liver were removed to avoid rapid putrefactive processes of these organs, which would affect the viability of *B. anthracis*. The carcass was kept for 36 h at room temperature to induce spore formation and was then transferred to the cage containing the flies. All flies were allowed to feed on the carcass. The flies were then divided into four groups, based on contact duration of the infective feed. After 2 h, the first group of 200 flies (Group A) was collected and transferred to Petri dishes. Thereafter, three other groups of 200 flies each (Group B, C, and D) were transferred to different Petri dishes after 4, 6, and 8 h, respectively. Flies were chilled to 4°C and then collected using an entomological net.

### Fly contamination by feeding on infected blood

For this experiment, the flies were housed as described previously. The flies were exposed to a cotton-wool pad, placed in the middle of the cage, saturated with 8 ml of rabbit defibrinated blood containing about 8×10^8^ spores of *B. anthracis* strain A0843. After 2, 4, 6, and 8 h following their infective feed, four groups of about 200 flies each, from Groups A–D (as previously mentioned) were transferred to Petri dishes.

### Fly spot bacteriological examination

Spots (feces and vomitus) from the flies of Groups A–D, deprived of food from the time of their capture, were tested for the presence of *B. anthracis.* Flies from each group were transferred to new Petri dishes every 2 h, and spots were collected with 10 ml of saline in 15 ml tubes at 4°C. The four suspensions containing spots were serially diluted in phosphate buffered saline (PBS, pH 7.2) and then plated in triplicate on Trimethoprim, Sulfamethoxazole, Polymixine (TSMP) agar added with 5% of sheep erythrocytes. The plates were incubated aerobically at 37°C for 48 h. Isolated colonies on the TSMP plates were initially identified as *B. anthracis* by colony morphology and Gram stain. To confirm anthrax, two colonies per plate were tested using specific PCR assays [15].

### Immunofluorescence assay detection of B. anthracis vegetative forms

To verify the ability of anthrax spores to germinate and replicate in the gut of flies, 20 specimens of *M. domestica*, housed as previously mentioned, were brought into contact with 8 ml of rabbit defibrinated blood containing 1.2×10^6^/ml of anthrax spores for 2 h. The blood was pre-treated by heating at 56°C for 30 min to eliminate any residual vegetative forms of *B. anthracis*. Every 30 min, the blood was replaced with fresh contaminated blood to avoid the germination of spores. After 2 h following the infective feed, the flies were killed and the gut content of each fly was removed aseptically and suspended in 500 µl of PBS. After centrifugation at 2000 rpm for 10 min, the suspensions were washed twice with PBS and inactivated with 1% formalin. These suspensions were then used as antigens in a direct Immunofluorescence Assay (IFA). As controls, two inactivated suspensions were prepared with *B. anthracis* virulent strain A0843 (positive control) and *B. anthracis* noncapsulated strain Sterne (negative control).

IFA was performed as follows: each well of a slide used for immunofluorescence (BioMèrieux) was coated with 20 µl of formalin-inactivated suspension. The slides were air-dried and then fixed for 30 min in ethanol (99%) at −20°C. After fixing, *B. anthracis* vegetative forms were detected with the specific monoclonal antibody F26G3 coupled to Alexa Fluor 488 (added to 5 µg/ml in each well), which binds the capsule of *B. anthracis* virulent strains [16]. The slides were incubated for 30 min at 37°C and were then washed thrice for 5 min in PBS. The last wash was performed with distilled water. After washing, the slides were mounted with Fluoprep (BioMérieux) and observed with a fluorescence microscope (Zeiss Axiostar Plus) at a magnification of 40×. The monoclonal F26G3 Alexa Fluor 488 was kindly supplied by Dr. Kozel [16].

### Anthrax spore replication in the guts of flies fed on different substrata

Four groups of 30 flies each were fed in separate Petri dishes as follows: Group A, defibrinated rabbit blood; Group B, 10% sugar solution; Group C, milk; and Group D, a mixture containing 46% powdered milk, 46% sugar, and 8% powdered eggs. Feeding was performed using a saturated cotton-wool pad. After 6 h, the flies were killed and the gut content of each fly was aseptically removed. The gut contents of flies belonging to the same group were pooled in a tube containing 10 ml of saline. The four suspensions, Groups A–D, were sterile-filtered (using a 0.2 µm filter), inoculated with ∼50 spores/tube of *B. anthracis* A0843, and then incubated aerobically at 37°C. At 2 h intervals, 100 µl of each suspension was plated in triplicate on TSMP plates and incubated as previously mentioned. The growth of *B. anthracis* was monitored for 12 h.

### Statistical analysis

The *Kruskal—Wallis* test, a nonparametric method, was used to compare the four independent fly Groups (A–D) with a significance cutoff value of 0.05. The normal Q—Q plot was used to estimate non-normality of the data.

## Results

### Detection of B. anthracis in infected fly spots

**Figure 1:**
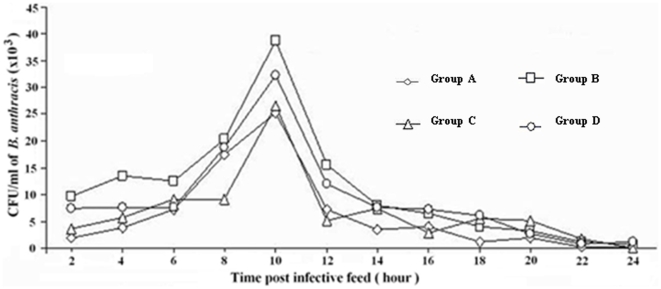
Number of CFU/ml of *B. anthracis* detected in fly spots. Flies were brought into contact with an anthrax-infected rabbit carcass for 2 h (Group A), 4 h (Group B), 6 h (Group C,) and 8 h (Group D), respectively. Spots were examined every 2 h, for 24 h after the infective feed. Data are means of 4 independent experiments.

Groups of *M. domestica* were fed on *B. anthracis*-infected rabbit carcasses for 2, 4, 6, or 8 h, and the ability of *B. anthracis* to grow from fly spots was evaluated every 2 h (Figure 1). Anthrax organisms were recovered from spots of all fly groups after 2 h but not 24 h or later after the infective feed. These data are derived from ∼200 flies selected on the basis of contact duration with the infected substrata, and are presented as the means of four independent experiments (Figure 1). Data obtained from Groups A–D, which represent the four durations of infective feed (2, 4, 6, and 8 h), were not statistically different (*P*>0.05). Among these groups, *B. anthracis* were recovered primarily from fly spots 2 to 12 h after the infective feed, with a peak at 10 h (Figure 1). At this time, an average of 35000 CFU/ml was recovered from the spots of each fly. The presence of *B. anthracis* in fly spots was reduced significantly after 12 h and was largely absent at 24 h. Each fly on average eliminated (vomited or excreted) 74000 to 133000 CFUs of *B. anthracis* within 24 h following the infective feed (Figure 1). Interestingly, similar results were obtained by testing flies fed on anthrax-contaminated rabbit blood (data not shown).

### Presence of vegetative forms of B. anthracis in the fly gut

The presence of vegetative forms of *B. anthracis* was determined for 20 gut specimens of *M. domestica*, derived from flies previously fed anthrax spores. Vegetative forms of *B. anthracis* were detected in the fly gut with a monoclonal antibody (F26G3) specific for the capsule of *B. anthracis* vegetative cells [16]. Capsule was detected in 7 of the 20 samples (data not shown). It is probable that samples with negative results had been excessively diluted before the IFA.

### Fly gut anthrax spore replication

**Figure 2:**
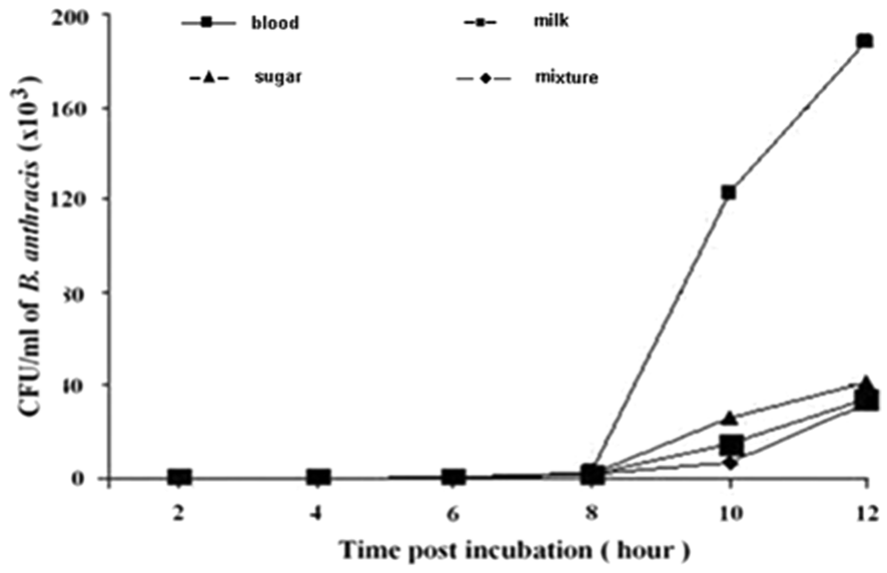
Number of CFU/ml of *B. anthracis* grown in 4 different media. The media consisted of a pool of the internal gut content of 30 flies fed, respectively, with blood, 10% sugar solution, milk or a mixture containing 46% powdered milk, 46% sugar and 8% powdered eggs. Each growth medium was initially inoculated with 50 anthrax spores and then the replication of spores was evaluated at 2, 4, 6 and 8 h after incubation at 37°C, by spreading 100 µl of each suspension on TSMP plates.

To determine if the replication of anthrax spores in the gut content of flies was influenced by the *ingesta*, four groups of 30 flies each were fed different substrata. Defibrinated rabbit blood positively influenced the replication of anthrax spores compared to the other substrata (Figure 2). Bacteria were detected in blood after 8 h of culture and there was peak recovery of 1.2–1.8×10^5^ CFU/ml of *B. anthracis* between 10 and 12 h of culture (Figure 2). In contrast, spores that were cultured in the gut content of the flies that were previously fed with 10% sugar solution, milk, and a mixture containing 46% powdered milk, 46% sugar, and 8% powdered eggs, had a much lower bacterial replication. This experiment was repeated four times and similar results were obtained.

## Discussion

Anthrax outbreaks are usually limited both geographically and temporally, occurring as a few cases in restricted areas over a period of weeks to months, without spreading to distant areas. However, anthrax sometimes manifests characteristics of an epidemic [4]. During an anthrax epidemic, many factors are implicated in the spread of the infection [17]. *B. anthracis*-infected carcasses represent the major source of replicating anthrax cells, vegetative organisms, and spores. *B. anthracis* vegetative cells in an intact carcass are normally destroyed within 48–72 h by putrefactive processes that occur in the internal anaerobic environment. However, when the carcass is opened prior to this process, the vegetative cells of *B. anthracis* may escape into the aerobic environment. The opening of the carcass promotes the drying of tissues and aerosolization of body fluids, both of which can introduce vegetative cells of *B. anthracis* into nutrient-depleted microenvironments, where they can subsequently undergo sporulation [18], [19], [20].

In nature, carcasses are rarely left undisturbed for periods long enough for putrefaction to eliminate all vegetative cells. Instead, scavengers open the carcasses, inducing sporulation of *B. anthracis* cells. Many vectors are implicated in spore dissemination, such as carnivores that are less susceptible to anthrax than herbivores and avian scavengers. Furthermore, the potential role of insects as vectors has been supported by strong experimental evidence showing that they can contribute to the transmission of spores from animal to animal and from animal to vegetation [5], [21], [22].

Cutaneous cases in humans have been associated with insect bites in both Zimbabwe [23], [24], [25], [26] and India [27], [28]. The ability of several species of *Hematophagous diptera* to transmit *B. anthracis* mechanically for at least 4 h after contact with an infected animal, and the ability of other diptera to contaminate surfaces with *B. anthracis* either with spores in their feces or by direct contamination with spores or vegetative forms present on their body surfaces, has also been demonstrated [21], [27], [28].

The house fly, *M. domestica,* is considered to be a potential agent for disease transmission. The habitual movement of house flies from filthy substrata, such as human feces, animal excreta, carcasses, and garbage, to food makes them ideal candidate vectors for disease transmission. Such diseases may include cholera, shigellosis, salmonellosis, and others [29], [30]. Bacteria have been isolated from feces, vomitus, external surfaces and internal organs of house fly samples [7]. Kelly [31] and Thomas [32] demonstrated that the fecal–oral route is one of the bacterial transmission modes facilitated by flies. Structurally, the fly is well suited in picking up pathogens. Each of the six legs of the fly has hairy structures and pads that secrete a sticky material, thus adding to its pathogen transmission potential. Bacteria have been known to exist on the exterior surface of the flies [33] and more than 100 species of pathogenic organisms have been isolated from the digestive tract of flies [34]. These organisms survived for an appreciable length of time [35]. Flies swallow liquid food and usually regurgitate ingested material to liquefy solid materials, which facilitates digestion. Droplets of feces may be deposited during the feeding process. The deposition of fly excreta is known to contribute to their ability to spread bacterial infection [7].

Results of this study indicate that the duration of feed did not influence the contamination of flies with *B. anthracis* and, consequently, the elimination of anthrax cells. This is in agreement with previous studies showing that only a few minutes of feeding are needed for flies to become infected after contact with anthrax-infected carcasses or living animals. In particular, when flies were allowed to feed on live anthrax-infected goats, in which *B. anthracis* were present in blood, only 2–3 min were needed for *S. calcitrans* to obtain a full meal of blood. In this case, the 1250 to 1–2 million anthrax bacilli were recovered from each infected fly [5]. The time elapsed between the detection of anthrax bacilli in the blood and the death of the animal is frequently 5–8 h or more, which is sufficient for the flies to feed. Consequently, the exposure time to an infected carcass is the crucial factor for the contamination of flies.

In this study, anthrax spores were evaluated for their ability to germinate and replicate in the gut of the insects, in which the pH is 6 to 7.5 and therefore compatible with *B*. *anthracis* growth. Vegetative forms of *B. anthracis* were found in the gut of 7 out of 20 samples of *M. domestica*. The midgut of insects such as *M. domestica* often contains a noncellular semipermeable membrane, the peritrophic matrix (or peritrophic membrane, PM), which separates the contents of the gut lumen from the digestive epithelial cells lining the midgut. The PM is facilitates the digestive process in the insect gut and provides defense against infection by viruses and parasites [36]. Thus, the PM may have caused an underestimation of *B. anthracis* vegetative cells in the IFA. The presence of vegetative *B. anthracis* in the gut of *M. domestica* indicates that the gut content of flies represents a favorable habitat for the germination and replication of anthrax spores.

In conclusion, the results suggest that blood made optimal *pabulum* for anthrax (Figure 2). However, it cannot be excluded that the results of these experiments might be different at later times after feeding, e.g., between 12 h and 24 h post-feeding. Data from this study confirm that insects, in particular house flies, can mechanically transmit *B. anthracis*. Preliminary results suggest that the gut content offers a favorable anthrax habitat for spore germination and replication, especially when flies were fed blood. Further experiments are necessary to confirm these results.

In this study, the contact duration of flies with the infected carcass did not influence the number of anthrax cells eliminated (excreted or vomited), confirming that only a few minutes are required for fly contamination. The exposure time of infected carcasses to flies is especially important to consider during anthrax outbreaks in which there are a great number of deaths and infected animals. Athough the role of insects in the Basilicata anthrax epidemic remains unproven and is a hypothesis, fly control should be considered as a part of an anthrax control program, along with appropriate measures to promptly eliminate infected animals and carcasses.
